# Dishevelled PDZ domain targeting peptides modulate non-canonical Wnt5a/Ror signaling

**DOI:** 10.1016/j.bbrep.2026.102609

**Published:** 2026-04-30

**Authors:** Andrew C. Jubintoro, Ho-Jin Lee, Hsin-Yi Henry Ho, Jie J. Zheng

**Affiliations:** a Jules Stein Eye Institute, Department of Ophthalmology, David Geffen School of Medicine, University of California, Los Angeles, CA, USA; b Division of Natural and Mathematical Sciences, LeMoyne-Owen College, Memphis, TN, USA; c Department of Cell Biology and Human Anatomy, School of Medicine, University of California, Davis, CA, USA; d Molecular Biology Institute, University of California, Los Angeles, CA, USA

**Keywords:** Non-canonical Wnt, Wnt5a, Ror2, Dishevelled, Intramolecular interaction, PDZ, Kif26b

## Abstract

Dishevelled (Dvl) is a highly conserved scaffolding protein that plays a key role in the non-canonical Wnt signaling pathway. However, the exact mechanisms by which Dvl modulates pathway activation are not fully understood. The C-terminus of Dvl binds intramolecularly to its PDZ domain, stabilizing Dvl in an autoinhibited, closed state. To evaluate the importance of this intramolecular interaction, we used PDZ-binding peptides to disrupt the autoinhibitory binding. We found that these peptides activated non-canonical Wnt5a/Ror signaling. However, this effect was weaker than that of Wnt5a, suggesting that other factors likely contribute to the full signaling response. Additionally, these PDZ-binding peptides affected Wnt5a-induced signaling, suggesting the significance of Dvl's intermolecular interactions in determining signaling outcomes. Given the wide range of intracellular proteins that interact with the Dvl PDZ domain, these proteins can regulate Wnt signaling by controlling the transition from an inactive, closed state to an open, and possibly active, state.

## Highlights

• Dishevelled (Dvl) intramolecular interaction modulates its activity in non-canonical Wnt signaling.
• Disruption of Dvl intramolecular interaction activates non-canonical Wnt signaling.
• Dvl PDZ-binding peptides potentiate Wnt5a-induced signaling activity.
• Endogenous PDZ-binding proteins may regulate Wnt signaling by modulating the intramolecular interaction of Dvl.

## Graphical abstract

**Figure:**
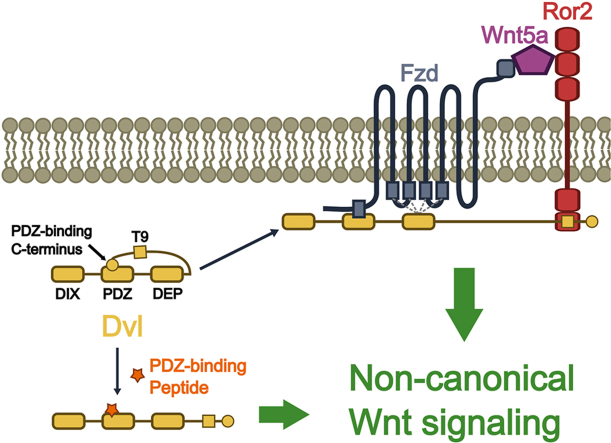

## 1 Introduction

The Wnt signaling pathways comprise a set of evolutionarily conserved pathways critical to numerous developmental processes. Dysregulation of these pathways can lead to congenital birth defects, developmental abnormalities, and cancer [1]. Wnt signaling is broadly categorized into the canonical β-catenin-dependent pathway and the non-canonical β-catenin-independent pathways. The selection of a specific pathway depends primarily on the relative usage/concentrations of Wnt ligands, antagonists, and receptors [2].

Dishevelled (Dvl) is a family of scaffolding proteins that play an essential role in integrating and relaying Wnt signals from the cell membrane to distinct downstream signaling cascades [[3], [4], [5]]. Dvl proteins are highly conserved and structurally composed of three modular domains—the DIX, PDZ, and DEP domains—connected by three intrinsically disordered regions (IDR1-3), with a disordered C-terminal region (Fig. S1) [6,7]. The PDZ domain mediates protein-protein interactions between Dvl and proteins bearing a PDZ-binding motif and has been implicated in both canonical and non-canonical Wnt signaling, though its role in the former remains controversial. Early studies demonstrated that the PDZ domain binds a conserved KTxxxW motif in the cytoplasmic tail of Frizzled (Fzd), an interaction proposed to be essential for canonical Wnt signal transduction [8]. However, subsequent complementation studies challenged this view, showing that the PDZ domain is, in fact, dispensable for canonical signaling [9,10]. In contrast, its contribution to non-canonical signaling is better supported. For instance, the Dvl PDZ domain binds and activates WGEF by releasing it from its autoinhibitory state, promoting Wnt/PCP signaling [11]. Additionally, the PDZ-binding protein Daple promotes non-canonical Wnt/Rac signaling by enhancing the association of Dvl with atypical protein kinase C, thereby facilitating Rac activation [12].

Notably, the Dvl PDZ domain also participates in an autoinhibitory interaction in which the extreme C-terminus of Dvl binds its own PDZ domain, stabilizing a closed, autoinhibited conformation [13]. While this autoinhibitory interaction does not appear to affect canonical Wnt signaling, the autoinhibited state of Dvl is markedly less active in non-canonical signaling. The transition to an open conformation potentiates non-canonical Wnt signaling, as evidenced by pronounced planar cell polarity phenotype in *Xenopus laevis* and *Danio rerio* models [13,14]. Together, these findings suggest that conformational switching of Dvl is a critical regulatory mechanism in non-canonical Wnt signaling, with the open state representing a key activation step. In this study, we sought to build on this model by investigating whether this intramolecular regulation similarly governs non-canonical signaling in the context of endogenous Dvl proteins.

Among the non-canonical Wnt signaling pathways, the Wnt5a/Ror pathway is one of the best characterized, regulating cytoskeletal dynamics to drive cell rearrangements and tissue morphogenesis [15]. Pathway activation is initiated upon the formation of a Wnt5a, Fzd, and receptor tyrosine kinase 2 (Ror2) complex at the plasma membrane, with Dvl serving as a critical intermediary that transduces the signal from this receptor complex into the cytoplasm [16]. The physiological importance of this pathway is underscored by the association of mutations in Dvl with Robinow syndrome [[17], [18], [19], [20]], a rare skeletal dysplasia also caused by mutations in other Wnt5a/Ror pathway components, including Wnt5a [21], Ror2 [22], and Fzd2 [23]. One downstream consequence of Wnt5a/Ror activation is the ubiquitin/proteasome-mediated degradation of the kinesin superfamily protein Kif26b [24], a response that can be exploited as a quantitative readout of pathway activity using GFP-tagged Kif26b expressed in live cells serving as a reporter of Wnt5a/Ror signaling [25].

With this assay, we found that Dvl PDZ-binding peptides activated Wnt5a/Ror signaling in live cells, even in the absence of exogenous Wnt5a, though the maximal activation achieved was lower than that elicited by Wnt5a itself. We further showed that these peptides modulate Wnt5a-induced signaling in an affinity-dependent manner. Given the diverse array of intracellular Wnt-signaling modulating proteins that interact with the Dvl PDZ domain, our findings suggest that these binding partners may play a key role in non-canonical Wnt signaling by possibly regulating the intramolecular shift of Dvl between its closed and open forms.

## 2 Materials and methods

### 2.1 Peptide synthesis and purification

Peptide pen-N3 was purchased from Millipore Sigma (Cat#322337). Peptide pen-DprWW was chemically synthesized, purified by reverse-phase high-performance liquid chromatography, and lyophilized by the Hartwell Center for Bioinformatics & Biotechnology at St. Jude Children's Research Hospital.

### 2.2 Canonical Wnt/β-catenin assay

HEK 293 STF cells were cultured in DMEM (Thermo Fisher Scientific, Cat#11965092) supplemented with 10% FBS (VWR, Cat#97068-085), 1X MEM NEAA (Thermo Fisher Scientific, Cat#11140050), 10 mM HEPES, and 200 μg/mL Geneticin (Thermo Fisher Scientific, Cat#10131027) at 37 °C/5% CO_2_ in a humidified incubator. Cells were seeded onto a clear-bottom, black 96-well plate at 5 × 10^4^cells/well and incubated overnight. Cells were pre-treated with Dvl PDZ peptides in 0.5% DMSO for 30 min before the addition of 0 or 200 ng/mL Wnt3a (Bio-Techne, Cat#5036-WN) upon which cells were incubated for 18 h. Cell viability and firefly luciferase activity were measured using the ONE-Glo + Tox Assay (Promega, Cat#E7120) following the manufacturer's protocol. Wnt3a was dissolved in DPBS with 0.1% BSA. Aggregate fluorescence and luminescence measurements of cells plated in n = 3 concurrent cultures were taken with the SpectraMax ID3 plate reader. Wnt pathway activity was calculated as fold-change luminescence values of each sample relative to vehicle control. Dose-response curves were fitted with a four-parameter log-logistic function with the drc package in R. IC_50_ estimates along with confidence intervals can be found in the results section.

### 2.3 Non-canonical Wnt5a/Ror assay

GFP-Kif26b NIH/3T3 cells were cultured in DMEM supplemented with 10% FBS and 1X Antibiotic-Antimycotic (Thermo Fisher Scientific, Cat#15240062) at 37 °C/5% CO_2_ in a humidified incubator. Cells were seeded onto a 48-well plate at 5 × 10^4^ cells/well and incubated for 18 h. Cells were pre-treated with Dvl PDZ peptides in 0.5% DMSO for 30 min before the addition of 0 to 1000 ng/mL Wnt5a upon which cells were incubated for 6 h. Cells were then dissociated with 0.25% Trypsin-EDTA and neutralized with growth medium. Cells were pelleted by centrifuging at 12,000×*g* at 4 °C for 3 min and resuspended in DPBS with 0.5% FBS. Flow cytometry was performed on a Sony MA900 Cell Sorter. Wnt5a (Bio-Techne, Cat#645-WN-010) was dissolved in DPBS with 0.1% BSA and 0.5% w/v CHAPS. Gating of live cell population via side scatter and forward scatter was performed on floreada. io. Non-canonical Wnt activation was measured through the percent downregulation of median GFP fluorescence: (GFP_control_ – GFP_treatment_)/GFP_control_ × 100%. 95% confidence intervals for median GFP downregulation were calculated from individual cell measurements obtained from n = 2 concurrent cultures using bootstrapping implemented with the boot package in R. Dose-response curves were fitted with a four-parameter log-logistic model using the drc package in R. Parameter estimates can be found in Table 1.

**Table 1:** Dose-response curve parameter estimates for non-canonical Wnt5a/Ror assay.

Condition	Minimum (%)		Maximum (%)		EC_50_ (ng/mL)		EC_50_ Difference (ng/mL)		
	Estimate	SE	Estimate	SE	Estimate	SE	Estimate	SE	p-value
pen-N3	−2.13	1.05	17.89	2.35	7.25	0.35	–	–	–
pen-DprWW	0.58	0.86	26.34	1.77	4.40	0.40	–	–	–
Wnt5a - pen-N3	−0.32	1.29	48.87	2.31	96.23	8.77	39.21	11.17	0.0029∗∗
Wnt5a + pen-N3	14.78	1.90	50.57	1.71	57.02	6.92			
Wnt5a - pen-DprWW	−0.24	1.12	52.05	1.91	108.07	9.55	−11.06	20.50	0.5969
Wnt5a + pen-DprWW	27.19	1.03	52.37	1.79	119.14	18.15			

Parameter estimates were obtained by fitting the percent median GFP downregulation for each dose-response series to a four-parameter log-logistic model with the drc package in R. Estimate: model parameter estimate, SE: standard error. Statistical testing for differences in EC_50_ was done with a two-tailed Student's t-test. ∗∗*p* < 0.01.

### 2.4 AlphaFold 3

The complex structure of mouse Dvl1 PDZ (Uniprot: P51141, N248-D340a) and peptide Dpr (SLKLMTTV), DprWW (SLKLMWWV), or N3 (EIVLWSDIPG) was predicted using AlphaFold 3 (AF3) server (https://alphafoldserver.com/, accessed on Jan. 7th, 2025). For N3 peptide, we added additional Gly residue at the C-terminus to mimic amidation (-NH_2_). The predicted structure of mDvl1 PDZ with peptide N3 was compared with the reported X-ray structure (PDB ID: 3CC0) [26], resulting in 0.639 Å of backbone RMSD, supporting the overperformance of AF3. The accuracy of the complex structures was also confirmed by the pLDDT and ipTM values. No template was used during the calculation. AF3 generated five models for each complex. We used a top-ranked complex model structure to calculate the Gibbs free energy change (ΔG in kcal/mol) of the complex structures using the VD-MM/GBSA (Molecular mechanics generalized Born surface area) method with HawkDock2 server (accessed on Sept. 12th, 2025).

### 2.5 UV absorption spectra of peptides

For consistency across experiments, we calibrated our peptide stock concentrations by measuring their UV spectra with a Nanodrop 2000 (Thermo Fisher Scientific). We used the following extinction coefficients for the peptides, determined by the number of tryptophan residues (5690 M^−1^cm^−1^ for each tryptophan): 22,760 M^−1^cm^−1^ for pen-DprWW and 17,070 M^−1^cm^−1^ for pen-N3. An example spectrum for each peptide can be found in Fig. S4. Note that we utilized the auto-pathlength feature of the Nanodrop, which automatically adjusts the pathlength to a shorter pathlength for high concentration samples. However, the presented absorbance is one that would be obtained if a 1 mm pathlength was used.

## 3 Results

### 3.1 DprWW peptide binds Dvl PDZ domain

PDZ domains typically bind short C-terminal peptides and are grouped into two domain classes, with their ligand motifs classified into three classes [[27], [28], [29]]. The Dvl PDZ domain is atypical as it does not fall into either domain class, has a larger binding pocket than canonical PDZ domains, and is highly promiscuous. One of the earliest identified Dvl-binding partners, the C-terminal peptide of Dapper [30], is a class I motif, yet it can also engage class II motifs [28,31] and even recognize Dvl's own class III-type C-terminus [8,13,14]. Numerous additional proteins have since been shown to interact with the Dvl PDZ domain, highlighting its unusual versatility [4,7].

The C-terminus of the Dvl PDZ-binding Dapper protein (SLKLMTTV) terminates in the PDZ-binding motif MTTV [30]. In an effort to enhance its binding affinity, guided by previous computational work [31], we substituted the two threonine residues with tryptophan. Additionally, we fused a penetratin sequence [32] to the N-terminus of the modified peptide to facilitate its cellular internalization and designated this fusion peptide pen-DprWW (RQIKIWFQNRRMKWKKGSLKLMWWV). Due to the abundance of proteins containing PDZ domains in the cell [28], we also included another Dvl PDZ-binding peptide pen-N3 (Ac-RQIKIWFQNRRMKWKKGGGEIVLWSDIP-NH_2_) in this study to ensure specificity and minimal off-target effects [26,33]. Unlike Dapper, the N3 peptide lacks the typical C-terminus PDZ-binding motif. Instead, it interacts with the Dvl PDZ domain through an internal sequence.

Using AlphaFold3, we generated structural models of the Dvl1 PDZ domain in complex with Dpr, DprWW, and N3 (Fig. S2) [34,35]. The predicted complexes exhibited high confidence scores (ipTM/pTM): PDZ-Dpr (0.93/0.92), PDZ-DprWW (0.78/0.87), and PDZ-N3 (0.83/0.82), supporting the reliability of the modeled interfaces.

To compare relative interaction strengths, we performed MM/GBSA energy scoring using HawkDock2 [36]. The resulting binding energy estimates ranked the peptides as DprWW > N3 > Dpr, with calculated values of −85.81, −81.29, and −69.33 kcal/mol, respectively. Notice that these values represent computational energy scores within the MM/GBSA framework and should not be interpreted as experimentally determined standard-state free energies [36]. Rather, they are used here to compare relative binding propensities among the modeled complexes.

### 3.2 Dvl PDZ-binding peptides inhibit canonical Wnt/β-catenin signaling

**Fig. 1:**
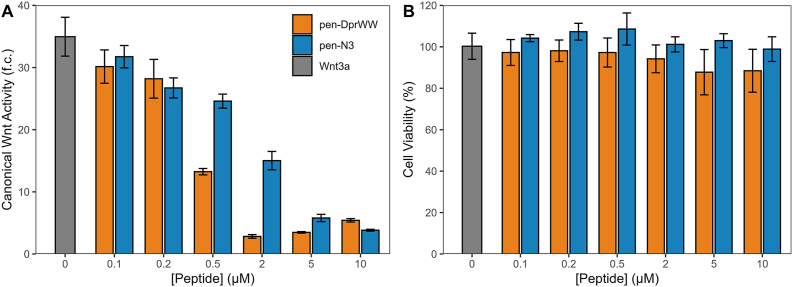
Dvl PDZ-binding peptides inhibit canonical Wnt/β-catenin signaling (A) Dvl PDZ-binding peptides pen-DprWW and pen-N3 inhibit 200 ng/mL Wnt3a-induced Wnt/β-catenin signaling as measured through the SuperTopFlash reporter in HEK 293 STF cells. Cells were pre-treated with 0-10 μM of the peptides for 30 min, followed by an 18 h incubation in peptide + Wnt3a media, after which luciferase levels were measured using the Promega ONE-Glo assay. The peptides pen-DprWW and pen-N3 inhibited signaling activity with IC_50_ values of 0.36 μM (95% CI: 0.29, 0.43) and 0.92 μM (95% CI: 0.58, 1.26), respectively. (B) The peptides displayed negligible levels of cytotoxicity as measured by the Promega CellTiter-Fluor assay. Data are presented as the mean ± SD from aggregate measurements of cells plated in n = 3 concurrent cultures.

To further confirm the activity of the two peptides, we quantified its ability to inhibit Wnt3a-induced canonical Wnt signaling using the TOP-Flash reporter assay (Fig. 1A). We found that pen-DprWW displayed micromolar levels of activity in inhibiting Wnt3a-induced signaling with a half-maximal inhibitory concentration (IC_50_) of 0.36 μM (95% CI: 0.29, 0.43). Similarly, as expected, pen-N3 inhibited Wnt3a-induced signaling with an IC_50_ of 0.92 μM (95% CI: 0.58, 1.26). Furthermore, we assessed the cytotoxicity of these two peptides using the CellTiter-Fluor assay. This assay uses a cell-permeant substrate that is cleaved by a protease only active in live cells to produce a fluorescenct product. Loss of membrane integrity results in the inactivation of the protease, making the fluroescence signal a reliable indicator of cell viability. We found that at concentrations ranging from 0 to 10 μM, pen-DprWW and pen-N3 caused no significant decrease in cell viability, indicating negligible cytotoxicity (Fig. 1B).

### 3.3 Dvl PDZ-binding peptides activate non-canonical Wnt5a/Ror signaling

We then investigated the effects that the Dvl PDZ binding peptides pen-N3 and pen-DprWW have on the non-canonical Wnt5a/Ror signaling pathway by using an NIH/3T3 cell line stably expressing a GFP-Kif26b construct. This protein construct undergoes ubiquitin/proteasome-mediated degradation in response to Wnt5a/Ror pathway activation, allowing for a quantitative measure of non-canonical pathway activity [25].

**Fig. 2:**
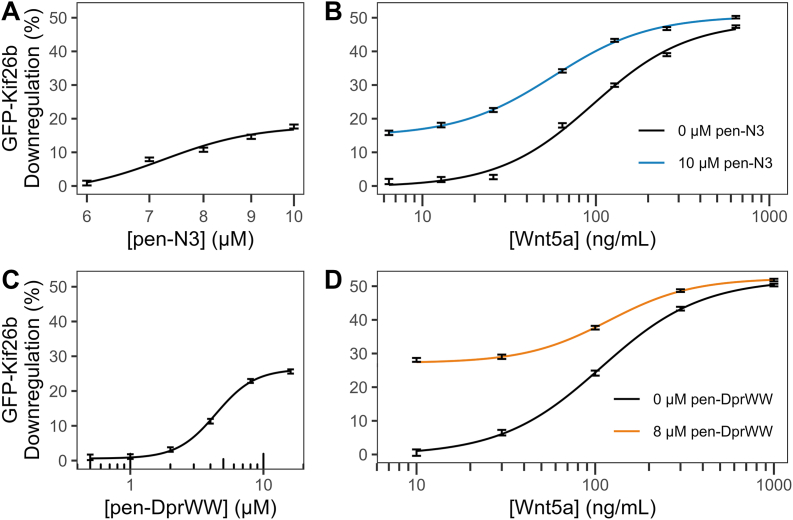
Dvl PDZ-binding peptides activate non-canonical Wnt5a/Ror signaling NIH/3T3 cell line stably expressing a GFP-Kif26b construct was treated with varying concentrations of Dvl PDZ-binding peptides for 6 h, followed by quantification of GFP downregulation through flow cytometry. The peptides pen-N3 (A) and pen-DprWW (C) activated non-canonical Wnt5a/Ror signaling with EC_50_ values of 7.25 μM (95% CI: 6.51, 8.00) and 4.40 μM (95% CI: 3.55, 5.26), respectively. Both pen-N3 (B) and pen-DprWW (D) cooperate with Wnt5a to enhance pathway activation. However, pen-N3 interfered with Wnt5a, reducing its EC_50_ by 39.21 ± 11.17 ng/mL (p = 0.0029), whereas pen-DprWW had no significant effect, increasing Wnt5a EC_50_ by 11.06 ± 20.50 ng/mL (p = 0.5969). Data points represent median GFP downregulation with error bars representing 95% confidence intervals from individual measurements of cells plated in n = 2 concurrent cultures.

To assess the extent to which the Dvl PDZ-binding peptides activate the non-canonical pathway, we treated the GFP-Kif26b reporter cells with varying concentrations of pen-DprWW and pen-N3. The amount of GFP fluorescence emitted by individual cells was quantified through flow cytometry, where its downregulation indicates Wnt5a/Ror pathway activation (Fig. 2). We found that these peptides activate the pathway in a dose-dependent manner, causing a sigmoidal-shaped downregulation of GFP fluorescence in relation to the concentration of peptides used (Fig. 2A and C). The peptides achieved half-maximal downregulation of GFP-Kif26b (EC_50_) at concentrations of 4.40 μM (95% CI: 3.55, 5.26) for pen-DprWW and 7.25 μM (95% CI: 6.51, 8.00) for pen-N3. To rule out non-specific effects that could be elicited from the penetratin sequence, we assayed a peptide made up of the penetratin sequence fused to a string of glycine and serine residues and found that this peptide did not activate the non-canonical Wnt5a/Ror pathway (Fig. S3).

Furthermore, we tested the effects that these peptides have when used in conjunction with exogenous Wnt5a. To accomplish this, we pre-treated the reporter cells with either pen-DprWW or pen-N3 at concentrations that elicited maximal pathway activation, 8 μM for pen-DprWW and 10 μM for pen-N3, then added varying concentrations of Wnt5a (0 to 1000 ng/mL). We found that these peptides interfere with Wnt5a to activate the pathway across the entire range of tested Wnt5a concentrations (Fig. 2B and D). The EC_50_ of Wnt5a in inducing GFP downregulation is 103.57 ng/mL (95% CI: 89.31, 117.84). In the presence of pen-N3, this EC_50_ is reduced to 57.02 ng/mL (p = 0.0029), whereas no statistically significant difference was observed in the case of pen-DprWW, where the EC_50_ remained at 119.14 ng/mL (p = 0.5969) (Table 1). When administered individually, the peptides achieved a maximal downregulation of GFP-Kif26b by about 15% and 25% for pen-N3 and pen-DprWW, respectively. This is about half of the maximum effect we observed when using Wnt5a, which was about 50% GFP-Kif26b downregulation. Notably, this maximum downregulation of GFP-Kif26b was achieved by 1000 ng/mL Wnt5a, with or without the addition of the Dvl PDZ-binding peptides.

## 4 Discussion

In this study, we demonstrated that Dvl PDZ-binding peptides are sufficient to activate Wnt5a/Ror signaling in live cells and that they modulate Wnt5a-induced signaling in an affinity-dependent manner. These findings build on prior work implicating PDZ-domain-mediated autoinhibition of Dvl in non-canonical signaling and supporting a regulatory role for endogenous PDZ-binding proteins in the Wnt signaling pathway. Taken together, our results are consistent with a model in which interactions between Dvl and intracellular PDZ-binding proteins regulate Wnt5a/Ror signaling, possibly by influencing the conformational equilibrium of Dvl between its closed, autoinhibited state and its open, active state—though further work will be needed to directly establish this mechanism.

**Fig. 3:**
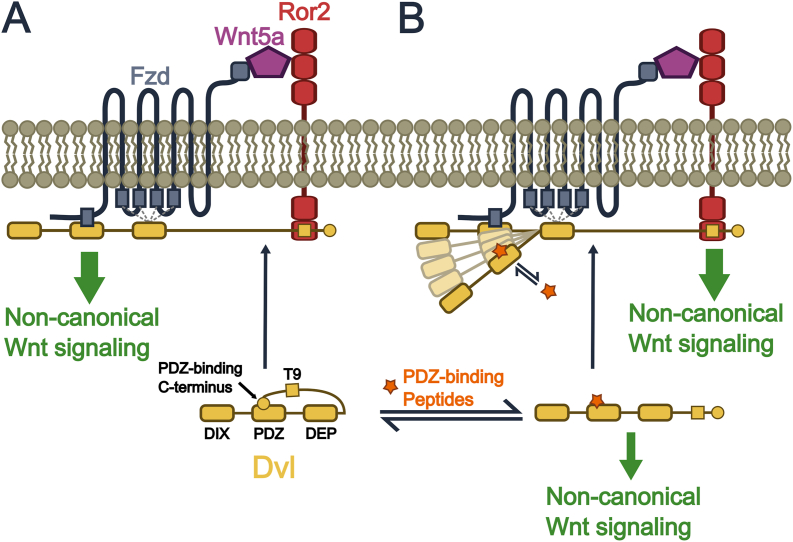
Proposed role of Dvl autoinhibition in regulating non-canonical Wnt signaling (A) Proposed model in which the transition of Dvl from a closed to an open conformation promotes its membrane recruitment, enabling interaction with the Wnt5a:Fzd:Ror2 complex and transduction of non-canonical Wnt signaling into the cytoplasm. (B) PDZ-binding peptides disrupt the autoinhibitory interaction of Dvl, promoting its open conformation and an increase in non-canonical Wnt signaling activity. However, this interaction is likely more complex in nature as while these peptides promote Dvl's active, open conformation, they would also disrupt the interaction between the Dvl PDZ domain and Fzd.

Protein autoinhibition is a widespread mechanism for regulating protein function [37]. In Dvl, its highly conserved C-terminal region intramolecularly binds to its PDZ domain, stabilizing a closed, autoinhibited conformation [13,14]. While the requirement for Dvl and its membrane recruitment in non-canonical signaling is well established [38,39], the mechanisms behind its recruitment and activation remain incompletely understood. Early studies suggested that the PDZ domain binds an internal KTxxxW motif within the cytoplasmic tail of Fzd, implicating this interaction in membrane recruitment. More recent work has pointed to the DEP domain [40] as the primary driver of membrane association, through interactions with negatively charged membrane surfaces [41] and with the cytoplasmic regions of Fzd [[42], [43], [44]]. In addition, a region 43 amino acids upstream of the C-terminal PDZ-binding motif, termed Tail 9 (T9), has been shown to associate with the cytoplasmic proline-rich domain of Ror2 [45]. Building on these findings and our prior work demonstrating a role for Dvl autoinhibition in membrane recruitment and non-canonical signaling activation, we propose a model in which relief of Dvl autoinhibition promotes Dvl association with the Wnt5a:Fzd:Ror2 membrane complex through engagement of its PDZ and DEP domains with Fzd and its T9 region with Ror2 (Fig. 3a).

Dvl autoinhibition is governed by the affinity of the C-terminus for the PDZ domain and the effective concentration of the tethered C-terminus, defined as the concentration of an identical freely diffusing C-terminus molecule required to displace half of the tethered ligand, and is often used to quantify this form of interaction [46,47].

However, our results show that effective concentration alone does not fully explain the states of Dvl autoinhibition observed in this study. If Dvl intramolecular interaction is modeled simply as competitive binding between the C-terminal region and free PDZ-binding peptides, one would expect that sufficiently high concentrations of free peptides would fully displace the intramolecular interaction between the C-terminus and the PDZ domain. However, using a live-cell reporter assay for non-canonical Wnt5a/Ror signaling, we observed a maximum response of only 30% to 50% of the effect elicited by Wnt5a, with the higher-affinity peptide producing the stronger response.

There are two possible explanations for those observations. First, the interaction between the tethered C-terminal region and the PDZ domain may be more nuanced than a simple ligand-receptor model, with the competition between the tethered C-terminus and free peptides influenced by their relative binding affinities. As a result, the fraction of Dvl molecules adopting the open state would partly depend on the affinity of the PDZ-binding peptides, with higher-affinity peptides leading to a greater maximal fraction of Dvl molecules in the open state, though the specific mechanism for this remains unclear. Second, in the complex environment of living cells, processes like degradation affect functional Dvl protein levels. It is possible that open-state Dvl is more susceptible to degradation, so low-affinity PDZ-binding peptides, which shift Dvl to the open state more slowly, would induce lower steady-state levels of active Dvl. Thus, the cellular response would depend not just on peptide concentration, but also on peptide affinity. In either case, this requires that Dvl binds much more tightly to the Wnt5a:Fzd:Ror2 complex than to the PDZ-binding peptides. We speculate that such augmented affinity is likely, in part, due to the multisite interactions between Dvl and the membrane complex [8,9,45] and other multivalent interactions [48,49] within the complex due to Fzd-Ror2 dimerization [16] induced by Wnt5a binding (Fig. 3A).

Moreover, despite these differences in maximal activation, we observed that Wnt5a and PDZ-binding peptides cooperate to activate non-canonical Wnt signaling. Notably, the lower-affinity peptide pen-N3 decreased the EC_50_ of Wnt5a, though this behavior was not observed for the higher-affinity pen-DprWW. This difference likely arises from the dual role of these peptides, as while they promote an open state of Dvl that facilitates membrane complex assembly, they also disrupt the Dvl-Fzd interaction (Fig. 3B). Nevertheless, our results demonstrate that their effects result in net pathway activation and that the affinity of a given PDZ-binding peptide influences how it modulates Dvl autoinhibition.

The Dvl PDZ domain interacts with a wide array of intracellular proteins that modulate Wnt signaling [3,7,50]. Like the peptides studied here, it is possible for these endogenous proteins to regulate non-canonical Wnt signaling by altering Dvl's conformational state. Such interactions can be fine-tuned through regulation of protein levels [51,52] and post-translational modifications to either Dvl [53] or its PDZ-binding partners [54]. Specifically, modifications to the PDZ domain or its ligand, and even the Dvl DEP domain linking the C-terminus to the PDZ domain, could shift the balance of Dvl intramolecular interaction. This regulated activation of non-canonical Wnt signaling, independent of Wnt ligands, may explain findings that PCP signaling in *Drosophila* wings can proceed in the absence of Wnt ligands [55,56]. Additionally, emerging evidence of crosstalk between the canonical and non-canonical pathways [24,57] offers an intriguing avenue to explore the extent to which Dvl's conformational shift during Wnt/β-catenin signaling contributes to non-canonical pathway activation. Altogether, this model suggests that intracellular modulation of Dvl conformation provides an additional regulatory layer that complements extracellular Wnt inputs, suggesting a conserved mechanism by which Dvl's intramolecular interaction integrates diverse signals to fine-tune non-canonical Wnt signaling.

Despite these findings, we acknowledge several limitations to the present study. First, given the large body of proteins containing PDZ domains, the effects elicited by the PDZ-binding peptides may be influenced by off-target interactions either through disruption of other Dvl PDZ-mediated interactions or through engagement with PDZ domains of unrelated proteins. Although the peptides used here were selected for their distinct binding modes to the Dvl PDZ domain (C-terminal versus internal ligand), which provides some support for the specificity of the observed effects, off-target contributions cannot be fully excluded. Second, the observed enhancement of non-canonical Wnt signaling remains an indirect readout of Dvl autoinhibition relief, and experiments more directly linking conformational changes in Dvl to signaling activation will be necessary to substantiate the proposed model. Third, although the peptides were conjugated to a penetratin sequence to facilitate intracellular delivery, the concentrations reported here reflect extracellular dosing, and future studies incorporating pharmacokinetic and stability analyses would be valuable. Fourth, the experiments presented were conducted under technical replicates, and additional independent biological replicates will be needed to further establish the robustness of these findings. Finally, the findings presented in this study are limited to the effects of Wnt5a/Ror signaling modulation in cell-based systems, and *in vivo* validation remains an important area for exploration.

Our previous work has shown that the intramolecular interaction between the PDZ domain of Dvl and its C-terminus does not measurably regulate canonical Wnt signaling under the conditions tested [13]. A possible explanation for the differential effects of PDZ-binding peptides on canonical and non-canonical Wnt signaling is that PDZ occupancy modulates Dvl function in a context-dependent manner. In canonical Wnt signaling, productive signaling requires coordinated assembly of Dvl with Frizzled and LRP6 at the plasma membrane, and PDZ engagement by exogenous peptides may interfere with optimal receptor-coreceptor complex formation or membrane recruitment, thereby attenuating β-catenin activation. Moreover, the organization and regulation of the Dvl-Axin/LRP6 signalosome are increasingly recognized as more complex and context-dependent than current canonical models fully capture [58,59]. In contrast, in the Wnt5a/Ror pathway, PDZ targeting may relieve an autoinhibitory constraint or stabilize a Dvl state that preferentially supports interaction with Ror-associated signaling components, thereby enhancing pathway output. We note that genetic studies have suggested that the PDZ domain is largely dispensable for canonical signaling [9]. However, acute peptide occupancy is not equivalent to domain deletion and may modulate complex assembly or localization without implying essentiality. Thus, our data indicate that PDZ engagement can influence signaling output in a context-dependent manner rather than establish a strict requirement for the PDZ domain. While speculative, this framework provides a self-consistent interpretation of how the same PDZ-binding peptides can inhibit canonical signaling while enhancing non-canonical Wnt5a/Ror signaling.

## Author contributions

Conceptualization, J.J.Z. and A.C.J.; Methodology, A.C.J. and H.L.; Investigation, A.C.J. and J.J.Z.; Writing—original draft, A.C.J. and J.J.Z.; Writing—review and editing, H.L., H.H.H., and J.J.Z.; Funding acquisition, J.J.Z.; Resources, H.H.H. and J.J.Z.; Supervision, J.J.Z.

## Funding sources

This work was supported by the 10.13039/100000002National Institutes of Health (R01EY028557 and P30EY000331) and by Research to Prevent Blindness.

## Declaration of competing interest

The authors declare that they have no known competing financial interests or personal relationships that could have appeared to influence the work reported in this paper.

## Data Availability

Raw data of the results presented in this study are available from the corresponding author on request.
