# Supplementary material for: Dishevelled PDZ domain targeting peptides modulate non-canonical Wnt5a/Ror signaling
Source: Biochem Biophys Rep. 2026 Apr 30;46:102609. doi: 10.1016/j.bbrep.2026.102609 (PMC13142005; doi:10.1016/j.bbrep.2026.102609)
Supplement: Multimedia component 1 [file mmc1.pdf]

Supplementary Materials

Dishevelled PDZ domain targeting peptides modulate non-canonical Wnt5a/Ror signaling

Andrew C. Jubintoro,[1] Ho-Jin Lee,[2] Hsin-Yi Henry Ho,[3] and Jie J. Zheng[1,4,5,*]

[1]Department of Ophthalmology, David Geffen School of Medicine at the University of California, Los Angeles, Los Angeles, CA, USA
[2]Division of Natural and Mathematical Sciences, LeMoyne-Owen College, Memphis, TN, USA
[3]Department of Cell Biology and Human Anatomy, School of Medicine, University of California, Davis, Davis, CA, USA.
[4]The Molecular Biology Institute at the University of California, Los Angeles, Los Angeles, CA, USA.
[5]Address: UCLA Stein Eye Institute, Department of Ophthalmology, David Geffen School of Medicine at UCLA, 100 Stein Plaza, Los Angeles, CA 90095
*Correspondence: jzheng@jsei.ucla.edu

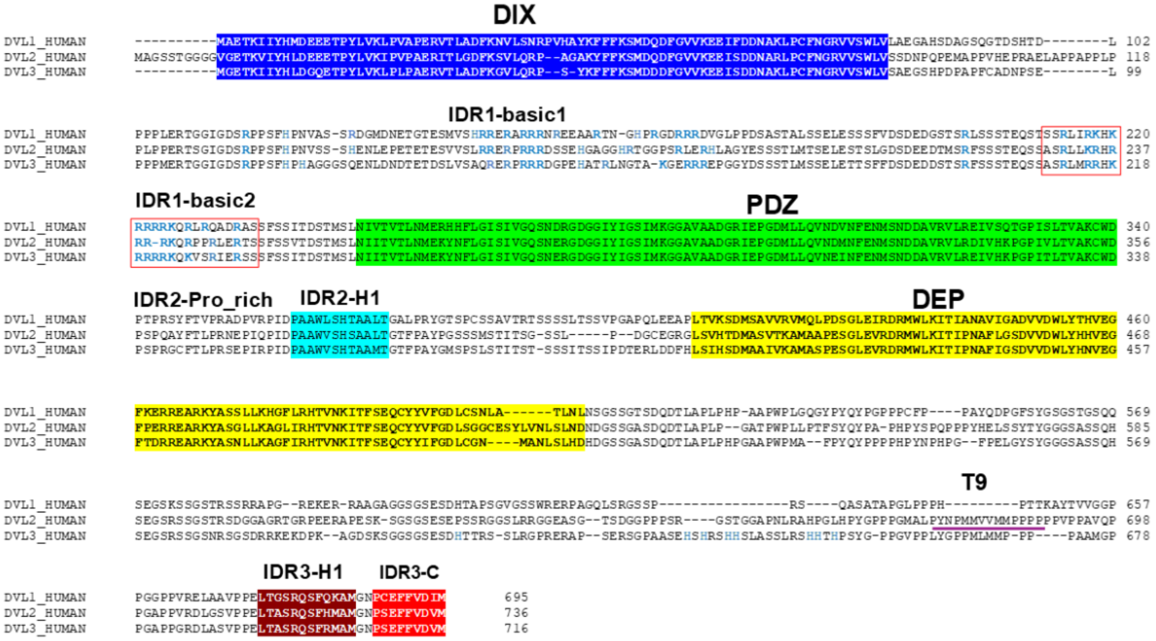

Figure S1. Sequence alignment of human Dvl proteins. Adapted from Fig. 2A of Wang et al., 2024 [1] with additional annotation of T9 region.

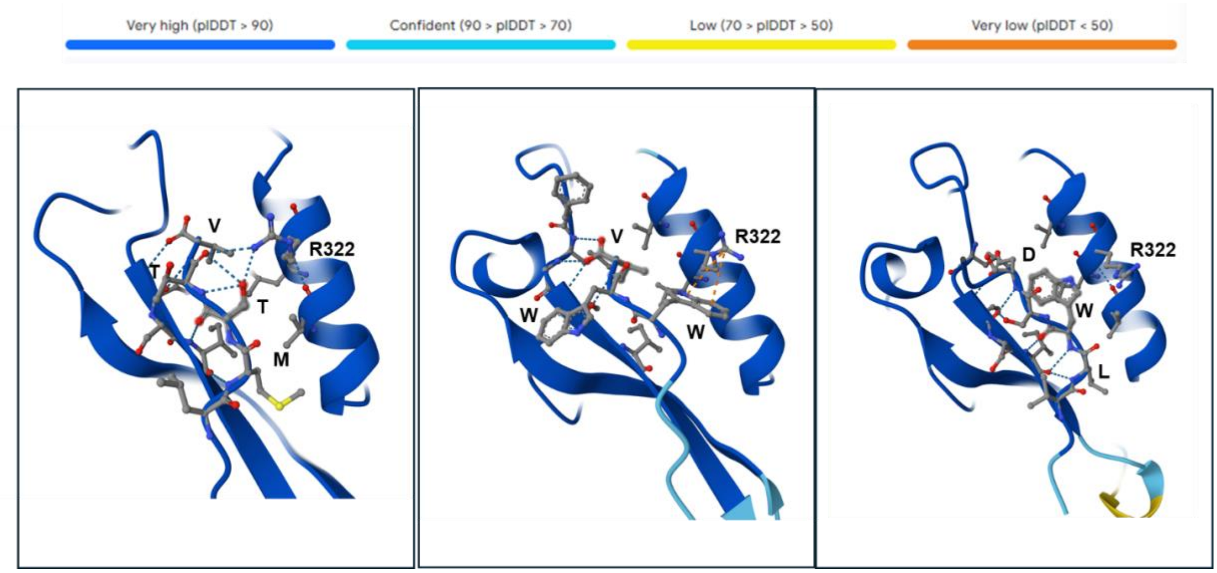

Figure S2. AlphaFold3-predicted structures of the Dvl1 PDZ domain bound to three different PDZ-binding peptides. Left, the Dpr peptide (SLKLMTTV); middle, the DprWW peptide (SLKLMWWV); and right, the N3 peptide (EIVLWSDIPG). Amino acid residues in the bound peptides at the binding interface are displayed in stick representation. The cartoon models are color-coded by predicted Local Distance Difference Test (pLDDT) values (scale shown above), with all three complexes rendered in blue, indicating uniformly high pLDDT confidence across the structures. Notably, comparison of the AF3-predicted Dvl1-N3 complex with the experimentally determined X-ray structure of the Dvl2 PDZ domain bound to the N3 peptide (PDB: 3CC0 [2]) yields a backbone RMSD of only 0.639 Å, demonstrating excellent agreement between prediction and experiment.

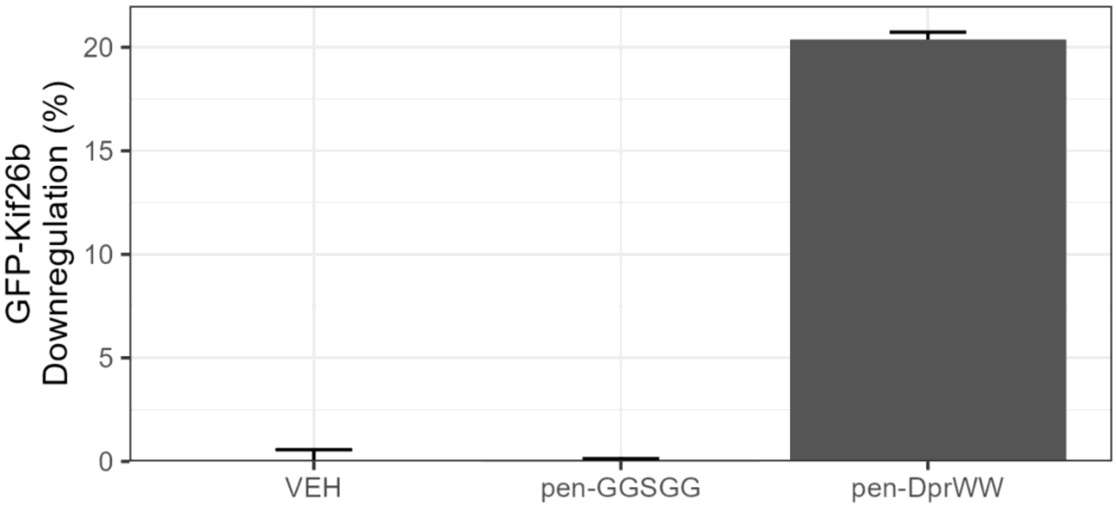

Figure S3. Dvl PDZ-binding-deficient peptide does not activate non-canonical Wnt5a/Ror signaling. NIH/3T3 cell line stably expressing a GFP-Kif26b construct was treated with vehicle control (1% DMSO), 8 µM pen-DprWW (RQIKIWFQNRRMKWKKGSLKLMWWV) or 7 µM pen-GGSGG (RQIKIWFQNRRMKWKKGGSGGSGG) for 6 h, followed by quantification of GFP downregulation through flow cytometry. The PDZ-binding peptide pen-DprWW activated non-canonical Wnt5a/Ror signaling, but the PDZ-binding-deficient peptide pen-GGSGG did not. Data points represent median GFP downregulation with error bars representing 95% confidence intervals.

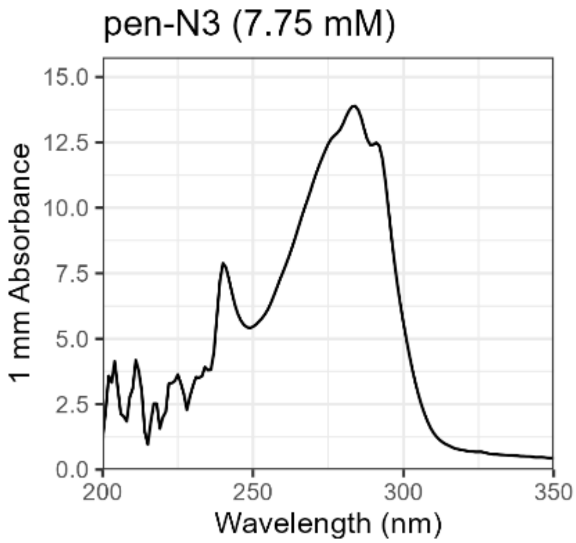

Figure S4. UV absorption spectra of pen-N3 and pen-DprWW. A_{280} of 7.75 mM pen-N3 = 13.227. A_{280} of 6.21 mM pen-DprWW = 14.13.

References

[1] L. Wang, R. Zhu, Z. Wen, H.-J.S. Fan, T. Norwood-Jackson, D. Jathan, H.-J. Lee, Structural and Functional Insights into Dishevelled-Mediated Wnt Signaling, Cells 13 (2024) 1870. https://doi.org/10.3390/cells13221870.

[2] Y. Zhang, B.A. Appleton, C. Wiesmann, T. Lau, M. Costa, R.N. Hannoush, S.S. Sidhu, Inhibition of Wnt signaling by Dishevelled PDZ peptides, Nat Chem Biol 5 (2009) 217–219. https://doi.org/10.1038/nchembio.152.
